# Editorial: Broadening the scope of addiction medicine: Integrating co-morbid conditions, polysubstance use, and patient experiences into substance use treatment

**DOI:** 10.3389/fpsyt.2022.1049420

**Published:** 2022-11-07

**Authors:** Matthew S. Ellis, Mance E. Buttram

**Affiliations:** ^1^Department of Psychiatry, Washington University in St. Louis School of Medicine, Saint Louis, MO, United States; ^2^Department of Health, Human Performance and Recreation, University of Arkansas, Fayetteville, AR, United States

**Keywords:** substance use disorder (SUD), substance use treatment, opioid use disorder (OUD), opioid treatment, addiction medicine, polysubstance use, integrated care approach

## Introduction

Addiction medicine is often siloed into treatment for a primary drug. However, low rates of treatment retention and success simultaneously occurring with increased rates of substance use disorders (SUDs), have led to calls to re-evaluate what it means to treat addiction. Importantly, there is a greater understanding that addiction exists in a feedback loop consisting of multiple factors such as social determinants of health, polysubstance use, and co-morbid mental and physical conditions. In addition, addiction medicine often confronts individual, organizational and structural barriers that prevent it from addressing these co-occurring issues, despite the fact they can directly impact treatment outcomes. The purpose of this issue was to highlight areas that can impact the development and treatment of substance use disorders, as well as ways that addiction medicine can be broadened by the development and implementation of integrated care, with the end goal of treating the whole person rather than a narrowed addiction, thus improving treatment outcomes for those who need it most.

## Polysubstance use and co-morbid conditions

Polysubstance use is a growing concern, including among people who use opioids, youth and young adults, and individuals with co-morbid conditions. Addressing these underlying issues, as well as identifying motivations to reduce substance use is paramount in meeting the goals of treatment for SUD. Co-morbid psychological or mental health problems are of special concern. One such example is borderline intellectual function (BIF), described by Hetland et al. The authors investigated the prevalence of BIF in Norway among individuals with poly-SUD. Results indicate that the number of patients with BIF is significant and it is associated with increased psychological distress among individuals engaged in poly-SUD treatment.

Several manuscripts in this issue note that identifying and treating underlying mental health co-morbidities can improve SUD treatment outcomes. First, findings from Gerhardt et al. show that among German patients in treatment for SUD, mental distress symptoms resulting from histories of childhood maltreatment (e.g., anxiety, depression, perceived stress) are associated with craving during treatment. Second, among rural government-financed health insurance beneficiaries in the United States (i.e., Medicare/Medicaid) who receive care at an opioid use disorder (OUD) treatment program, co-morbid anxiety, depression, and PTSD are common, as are stimulant and sedative use disorders (Lister et al.). And finally, utilizing a large-scale survey of American college students, Striley et al. examined the relationship between the use of vaping products and non-suicidal self-injury (NSSI). Findings documented a relationship between vaping, NSSI, suicidal ideation, and other substance use.

Together, these manuscripts highlight the need for additional screening and assessment of cognitive impediments, of which BIF is but one example, among individuals entering treatment for SUDs. Understanding the existence and severity of such impediments would allow treatment providers and clinicians to account for more tailored care management, as well as potential impacts (e.g., psychological distress) on treatment outcomes (Hetland et al.). Moreover, more comprehensive screening and assessment would likely indicate therapeutic targets for individuals with childhood maltreatment (Gerhardt et al.) and identify individuals who require additional support or integrated mental health care (Lister et al.; Striley et al.). Indeed, the recognition of the need to screen for co-morbid mental health and substance use disorders was the basis of the work of Shahzad et al. who translated and adapted the Cognitive Emotion Regulation Questionnaire into Urdu and tested it among a sample of Pakistani patients in treatment for SUD.

Understanding patient motivations is also critical for enhancing addiction medicine. As documented by Fortin et al., the desire to reduce one's consumption of illegal cannabis is a primary motivation for the use of cannabidiol products (CBD), especially among individuals who co-use alcohol and tobacco. CBD consumption was also found to reduce cannabis withdrawal symptoms.

## Patient experiences and integrated care

A key motive for this special issue on broadening the scope of addiction medicine was to highlight the urgent need for integrated care. Persons with SUDs suffer from a range of comorbid conditions and engage in a number of risk-laden behaviors that require treatment of the whole person in order to achieve desired treatment outcomes.

Several of the articles report on patient perspectives as a call to action to manage the multifaceted needs of persons with SUDs. Stoltman et al. report data from a medication for OUD (MOUD) clinic in West Virginia, noting that, outside of pregnant women, reproductive and sexual health (RSH) services are significantly lacking in OUD populations. While knowledge and uptake of contraceptives in both men and women was low, 40% indicated an interest in RSH services co-located with their MOUD clinic such as contraceptive counseling and provision, STI testing, and sexual dysfunction management. Stoltman et al. reinforce the benefit of co-location in rural areas, where it can be challenging to access multiple points of care.

Surratt et al. also report on patient perceptions of sexual health services in rural areas, specifically the barriers to initiating pre-exposure prophylaxis (PrEP) as a form of HIV prevention among persons who inject drugs (PWID) in southeastern Kentucky. HIV continues to disproportionately affect PWID, particularly in rural areas that experience distinct barriers. Individually, there were moderate perceptions of HIV risk, low awareness and knowledge of PrEP, and uncertainty about PrEP resulting from stigma from law enforcement and healthcare personnel, where concerns of privacy deterred PrEP-seeking behaviors. Layered on are structural barriers existing in the form of “PrEP deserts” where few to no qualified providers exist. Integrating PrEP services and education with existing syringe service programs would mitigate many barriers unique to PrEP-seeking in isolated rural areas.

While Stoltman et al. and Surratt et al. elucidated patient perceptions to highlight the benefits of integrated care, several articles reported data from programs that have implemented such care practices. Losikoff et al. note that cases of hepatitis C (HCV) have drastically increased in the United States. However, treatment initiation for HCV among PWID is low, at <10% among those screened. This may be due, in part, to a fear of stigma from healthcare professionals. As such, Losikoff et al. report on an outpatient OUD treatment center providing MOUD that developed a system to co-locate HCV services with MOUD, including screening, provider education, patient education, and treatment. Such integrated care led to HCV cure rates comparable to people who do not use drugs, lower rates of other substance use, and greater utilization and retention of OUD treatment.

Nolan et al. report on a bridge-to-health program in a Midwest academic hospital, developed for treating PWID that present with infections associated with injection drug use (IDU). To improve post-discharge outcomes related to both IDU and OUD, a multidisciplinary team provided patients receiving MOUD with infection-related care, harm reduction education and take-home kits, and follow-up care 90 days post-discharge, ending with a handoff to a community provider for continued addiction care. Qualitative interviews indicated that participants found the program beneficial for managing acute pain-related issues and improving access to MOUD, had positive perceptions of the multidisciplinary team, yet also noted issues surrounding hospital confinement and stigma from healthcare personnel, issues of important consideration in the implementation of integrated care.

Finally, Martin et al. raise the issue of poor sleep quality, a comorbidity of growing concern in SUD research and care. There may exist a bi-directional relationship between SUDs and sleep, wherein one may negatively impact the other. As part of a broader study seeking to classify individuals with SUD along neurofunctional domains, individuals with SUDs were compared to people who do not use drugs across sleep quality. Martin et al. report that poor sleep was more prevalent compared to controls in both men andwomen, and these findings were most robust for those with OUD or cannabis use disorder. The data also suggest that there are sex-specific factors, with poor sleep quality more prevalent among women, which may suggest that sleep dysfunction in individuals with SUDs may need to be addressed in sex or gender-specific ways.

## Future directions

Although examining different substance use populations and themes, the manuscripts in this issue highlight new areas of addiction medicine research and offer guidance for future directions. Specifically, the development of screening and assessment tools for treatment-seeking people who use drugs is critical, including translating existing tools into new languages. Such work allows for the integration of care to diagnose, treat, and prevent co-morbid mental health problems, and infectious diseases and address sleep quality and additional healthcare needs. Recognizing that addiction medicine needs to be more broadly defined, and addressing the barriers that exist in order to implement integrated or multidisciplinary care, are urgently needed in order to improve treatment outcomes and mitigate the substance use crisis that exists in the world today.

## Author contributions

All authors listed have made a substantial, direct, and intellectual contribution to the work and approved it for publication.

## Conflict of interest

The authors declare that the research was conducted in the absence of any commercial or financial relationships that could be construed as a potential conflict of interest.

## Publisher's note

All claims expressed in this article are solely those of the authors and do not necessarily represent those of their affiliated organizations, or those of the publisher, the editors and the reviewers. Any product that may be evaluated in this article, or claim that may be made by its manufacturer, is not guaranteed or endorsed by the publisher.

